# A-to-I RNA Editing: Current Knowledge Sources and Computational Approaches with Special Emphasis on Non-Coding RNA Molecules

**DOI:** 10.3389/fbioe.2015.00037

**Published:** 2015-03-25

**Authors:** Giovanni Nigita, Dario Veneziano, Alfredo Ferro

**Affiliations:** ^1^Department of Molecular Virology, Immunology and Medical Genetics, Ohio State University, Columbus, OH, USA; ^2^Department of Clinical and Molecular Biomedicine, University of Catania, Catania, Italy

**Keywords:** A-to-I RNA editing, ncRNA, microRNA, RNA-seq, ADARs, HTS

## Abstract

RNA editing is a dynamic mechanism for gene regulation attained through the alteration of the sequence of primary RNA transcripts. A-to-I (adenosine-to-inosine) RNA editing, which is catalyzed by members of the adenosine deaminase acting on RNA (ADAR) family of enzymes, is the most common post-transcriptional modification in humans. The ADARs bind double-stranded regions and deaminate adenosine (A) into inosine (I), which in turn is interpreted by the translation and splicing machineries as guanosine (G). In recent years, this modification has been discovered to occur not only in coding RNAs but also in non-coding RNAs (ncRNA), such as microRNAs, small interfering RNAs, transfer RNAs, and long non-coding RNAs. This may have several consequences, such as the creation or disruption of microRNA/mRNA binding sites, and thus affect the biogenesis, stability, and target recognition properties of ncRNAs. The malfunction of the editing machinery is not surprisingly associated with various human diseases, such as neurodegenerative, cardiovascular, and carcinogenic diseases. Despite the enormous efforts made so far, the real biological function of this phenomenon, as well as the features of the ADAR substrate, in particular in non-coding RNAs, has still not been fully understood. In this work, we focus on the current knowledge of RNA editing on ncRNA molecules and provide a few examples of computational approaches to elucidate its biological function.

## Background

While in the past researchers mainly focused on DNA mutations in order to further elucidate molecular pathways involved in numerous cancers, in the last decade focus has shifted to the analysis of post-transcriptional modification events, such as RNA editing. Concurrently, it has been estimated that only 1% of mammalian genome codes for protein, while the vast majority of the transcriptome is composed of non-coding RNAs crucially involved in gene expression pathways, such as transcription, translation, and gene regulation (Cech and Steitz, 2014). The editing machinery, occurring both in coding and non-coding RNAs, has been implicated in various human diseases (Galeano et al., 2012; Tomaselli et al., 2014). Strong interest is thus growing toward understanding how and why RNA editing can influence non-coding RNA function.

RNA editing is a type of post-transcriptional modification that takes place in eukaryotes. Several forms of RNA editing have been discovered, but nowadays A-to-I RNA editing is considered the predominant one in mammals (Nishikura, 2010). Adenosine (A) deamination produces its conversion into inosine (I), which in turn is interpreted as guanosine (G) by both the translation and splicing machineries (Rueter et al., 1999). Enzymes members of the adenosine deaminase acting on RNA (ADAR) family catalyze this biological phenomenon which occurs only on dsRNA structures (Bass, 2002; Jepson and Reenan, 2008; Nishikura, 2010). Double-stranded RNAs are imperfect duplexes formed by base-pairing between residues in the region proximate to the editing site (usually overlapping a neighboring intron) and the exonic sequence containing the A. Such proximate region is termed *editing complementary sequence* (ECS), potentially located several hundred to several thousand nucleotides upstream or downstream of the edited A. This requires experimental validation and represents one critical issue with the detection of editing sites.

Three members of the ADAR gene family can be distinguished in humans, in particular, two isoforms of ADAR1 (ADAR1p150 and ADAR1p110) (Kim et al., 1994), ADAR2 (Lai et al., 1997), and ADAR3 (Chen et al., 2000). While ADAR1 and ADAR2 are widely expressed in tissues, ADAR3 is limited to brain tissues (Melcher et al., 1996). Interestingly, unlike ADAR1 and ADAR2, ADAR3 possesses a catalytically inactive (Chen et al., 2000) arginine-rich R domain, which allows the enzyme to bind single strand structures.

An RNA edited site neighborhood profiling was established for ADAR1-2. While for ADAR1, no 3′ neighbor preference has been identified, a 5′ nearest neighboring preference consisting of U = A > C > G (Polson and Bass, 1994) can be observed. Like ADAR1, ADAR2 has a similar 5′nearest neighboring preference (U ≈ A > C = G) but, furthermore, it has a 3′nearest neighboring preference (U = G > C = A) as well, creating a particular trinucleotide sequence with the adenosine at the center (U*A*U, A*A*G, U*A*G, A*A*U) (Lehmann and Bass, 2000). In addition, the ADARs show selectivity based on both dsRNA length and the presence of mismatches, loops, and bulges that interrupt the base-pairing (Bass, 1997).

There are two kinds of A-to-I RNA editing: *specific* A-to-I editing occurs in short duplex regions interrupted by bulges and mismatches (Wahlstedt and Ohman, 2011); the *promiscuous* one occurs within longer stable duplexes of hundreds of nucleotides, mostly formed by repetitive elements, such as Alus, in which up to 50% of adenosines could be targeted by ADARs (Carmi et al., 2011; Bazak et al., 2014b).

Adenosine-to-inosine RNA editing has been discovered both in intronic and exonic regions, 5′ and 3′-UTRs as well. RNA editing events can take place in several cellular contexts: in the gene expression pathway (Bazak et al., 2014b), such as in translation (Nishikura, 2010) or in the creation and/or destruction of splicing sites (Rueter et al., 1999); during gene regulation through editing events in microRNA/mRNA binding regions (Nishikura, 2006; Borchert et al., 2009). Recent reports affirmed that RNA editing may occur in non-coding RNA molecules, particularly within precursor-tRNA (Su and Randau, 2011), pri-miRNA (Kawahara et al., 2008; Kawahara, 2012), and lncRNA (Mitra et al., 2012). It was estimated that 10–20% of miRNAs undergo A-to-I editing (Blow et al., 2006; Kawahara et al., 2008) at the pri-miRNA level (Yang et al., 2006). Editing can influence both the maturation process (Yang et al., 2006) and the recognition of binding sites on target mRNAs (Kawahara et al., 2007; Wu et al., 2011). Indeed, a single editing site in a miRNA seed region could drastically change its set of targets (Alon et al., 2012).

In the past decade, surprising results have been obtained in RNA editing site discovery, thanks initially to the application of bioinformatic approaches, subsequently fully replaced by RNAseq-based methods in recent years. The large amount of editing sites discovered by these methodologies has led to the creation of public databases (Kiran and Baranov, 2010; Kiran et al., 2013; Ramaswami and Li, 2014). As described below, all these resources containing very important information, such as editing level and genomic annotations, can help to functionally elucidate the RNA editing phenomenon.

This mini review summarizes both the current knowledge on RNA editing, as well as past and present approaches for discovery and analysis of editing sites, particularly emphasizing on RNA editing in non-coding RNA (ncRNA) molecules.

## Computational Approaches to Discover and Analyze RNA Editing Events

### The origins of the analysis and detection of RNA editing sites – computational and biochemical methods

In the early 2000s, the ADAR enzyme family was observed to play an important role during embryonic development (Higuchi et al., 2000; Wang et al., 2000), while also associating the alteration of the editing machinery to neurological diseases (Maas et al., 2001; Kawahara et al., 2004). At that time, only few RNA editing sites were discovered (Morse and Bass, 1999). Hoopengardner et al. (2003) using comparative genomics identified and experimentally validated 16 novel editing sites in fruit fly and one in human. Interestingly, they discovered that these editing sites are surrounded by highly conserved exonic regions which form a dsRNA structure as required for ADARs. Despite these efforts, most editing sites were detected by chance.

In 2004, unprecedented computational methods were designed in order to discover clustered A-to-I RNA editing sites in Alu repeats of the human transcriptome (Athanasiadis et al., 2004; Kim et al., 2004; Levanon et al., 2004), going from dozens to tens of thousands of editing sites. By aligning millions of publicly expressed sequence tags (EST) (Boguski et al., 1993) against a reference genome, it is indeed possible to identify A-to-G mismatches as putative candidates of A-to-I editing events. Unfortunately, without considering RNA editing, related features such as nearest neighbor preference sequence, this naïve approach produces a large amount of false positives due to sequencing errors originating from poor sequencing quality, somatic mutations, or single nucleotide polymorphisms (SNP). All of the above methods avoided this issue by taking into account cDNA-genome alignments along with clusters of mismatches in long and stable dsRNA structures and, finally, filtered known SNPs from the obtained candidates, reaching good accuracy.

A more quantitative and accurate analysis was later provided by Eggington et al. (2011)^1^, who predicted editing sites in dsRNAs by assuming a multiplicative relationship between the coefficients (estimated by a non-linear regression model and dependent on the bases neighboring each site) used to determine the percentage of editing sites.

The bioinformatics methods for RNA editing detection comparing a cDNA sequence with a reference genome nevertheless present a significant problem: they are not able to distinguish a guanosine originating from an I-to-G replacement, from a guanosine as a product of noise, sequencing errors, or SNP. To overcome this limit, Sakurai et al. (2010) designed a biochemical method, called inosine chemical erasing (ICE), for the identification of inosine sites on RNA molecules by employing inosine-specific cyanoethylation with reverse transcription, PCR amplification, and direct sequencing. Without requiring changing profiles of cellular gene expression nor genomic DNA for reference, this method accurately and consistently identifies inosines in RNA strands. Recently, Sakurai et al. (2014) combined the ICE method with deep sequencing technology (ICE-seq) for an unbiased genome wide screening of novel A-to-I editing sites.

### New *era* of RNA editing discovery – high-throughput sequencing approaches

Despite the substantial results achieved with the approaches described above, some restrictions due to sequencing limitations remained. Before 2009, in fact, only a few dozen editing sites had been detected outside repetitive regions in humans due to the impossibility of designing a systematic method to discover editing events in ncRNA genes.

With the advent of high-throughput sequencing technology (HTS), things radically improved. In 2009, Li et al. (2009) developed the first HTS-based application which, through massively parallel target capture and DNA sequencing, identified 36,000 non-repetetive putative A-to-I editing events. Recently, several HTS-based approaches for editing discovery have been developed (see Table 1). It was latterly hypothesized that there are more than 100 million editing sites in human Alu repeats, located mainly in genic regions (Bazak et al., 2014a). Despite the increased accuracy, these methods have limitations in terms of false positives produced (Kleinman and Majewski, 2012; Lin et al., 2012; Pickrell et al., 2012).

**Table 1:** **Deep sequencing based approaches**.

Focus	Year	# Editing sites (ES) discovered	Description	Reference
mRNAs	2009	239 A-to-I ES	Parallel target capturing and DNA sequencing	Li et al. (2009)
miRNAs	2010	10 (three A-to-I and two C-to-U)	Strategy to correct for cross-mapping in short RNA sequencing libraries	de Hoon et al. (2010)
mRNAs	2011	1,809 (1,096 A-to-I and 11 C-to-U)	Massively parallel DNA and RNA sequencing of 18 Korean individuals	Ju et al. (2011)
mRNAs	2012	9,636 (5,965 A-to-I)	Accurate mapping approach to distinguish single-nucleotide differences in one set of RNA-seq data	Bahn et al. (2012)
Coding, non-coding and small RNA genes	2012	22,588 (21,113 A-to-I)	Computational pipeline to identify RNA editing sites from genome and whole-transcriptome data of the same individual	Peng et al. (2012)
Alu and non-Alu regions	2012	150,865 (144,406 A-to-I) from GM12878	Framework to robustly identify RNA editing sites using transcriptome and genome deep-sequencing data from the same individual	Ramaswami et al. (2013)
		457,078 (423,377 A-to-I) from (Peng et al., 2012) data		
mRNAs	2012	61 A-to-I ES	Computational strategy based on two-step mapping procedure with only RNA-seq and without *a priori* RNA editing information	Picardi et al. (2012)
mRNAs	2012	5695 (5349 A-to-I)	A rigorous computational pipeline to identify RNA editing site in human polyA^+^ ENCODE RNA-seq data from 14 cell types.	Park et al. (2012)
miRNAs	2012– 2013	19 A-to-I ES	Protocol for the identification of RNA editing sites in mature miRNAs using deep sequencing data.	Alon et al. (2012) and Alon and Eisenberg (2013)
mRNAs	2013	>1 million of A-to-I ES in other human LCL and several tissues	Two methods (*separate* and *pooled* sample methods) to accurately identify RNA editing events by using RNA-seq data from multiple samples in a single species	Ramaswami et al. (2013)
mRNAs	2013	2,245 A-to-I ES	A strategy to accurately predict consecutive RNA editing events from human RNA-seq data in the absence of relevant genomic sequences	Zhu et al. (2013)
mRNAs	2013	223,490 A-to-I ES from (Ramaswami et al., 2013) data	Suite of python scripts to investigate RNA editing by using RNA-seq data	Picardi and Pesole (2013)
Alu elements	2014	1,586,270 A-to-I ES	Detection approach to analysis *Alu* editing by using large-scale RNA-seq data	Bazak et al. (2014a)
mRNAs	2014	29,843 A-to-I ES	Unbiased genome-wide screening of A-to-I editing events using the ICE-method combined with deep sequencing (ICE-seq)	Sakurai et al. (2014)
mRNAs	2014	455,014 A-to-I ES	Computational method to detect hyper-edited reads in RNA-seq data	Porath et al. (2014)

*Some of the most important deep sequencing based approaches, developed in the last 5 years, to identify RNA editing sites in humans*.

Table 1 depicts some of the most important studies on RNA editing detection by HTS. The majority was designed to identify RNA editing events in protein-coding RNA, while a few also focus on lncRNAs as well. In 2010, de Hoon et al. developed a strategy to correct cross-mapping of small RNA deep-sequencing libraries, applying it to analyze RNA editing in human mature miRNAs. They concluded that miRNA editing is rare in animals and addressed methodological problems in its analysis through RNAseq. Subsequently, Alon et al. (2012) systematically identified known editing events in mature miRNAs of human brain, in addition to 17 novel ones, 12 of which occur in the seed region (Alon and Eisenberg, 2013). They moreover identified sequence preference in the residues, both flanking and opposing the A-to-I editing site. As the authors suggested, this pipeline could identify editing sites in miRNAs from NGS data of different experimental set-ups. Currently, Alon’s method is the only one able to accurately detect and quantify A-to-I RNA editing events in mature miRNAs by NGS. Together with the latest pipeline published by Picardi et al. (2014) for RNA editing detection in human lncRNAs from deep sequencing experiments.

## Current Knowledge of RNA Editing on ncRNA Molecules

### Biological databases: DARNED and RADAR

The birth of the first computational methods for the identification of RNA editing events (Athanasiadis et al., 2004; Kim et al., 2004; Levanon et al., 2004) caused a growing interest in the scientific community for RNA editing, as there was a strong need to collect in a centralized repository the tens of thousands of editing events discovered up to that point. For this reason, Kiran and Baranov designed DARNED^2^ (DAtabase of RNa EDiting), the first public database of known editing sites in human (Kiran and Baranov, 2010). The first release of DARNED contained more than 40,000 predicted human editing sites, of which a few were experimentally validated (Ramaswami et al., 2013). The usefulness of the repository rests in the ability to retrieve information on RNA regions where editing events can occur, such as genome coordinates, cell/tissue/organ sources, and the number of ESTs supporting referenced and edited bases. According to the first release of DARNED, Laganà et al. (2012) built miR-EdiTar^3^, a database of predicted miRNA binding sites that could be affected by A-to-I editing sites occurring in 3′UTRs.

In subsequent years, the advent of high-throughput RNA sequencing (RNAseq) and biochemically-based (Sakurai et al., 2010) techniques progressively led to the development of increasingly accurate transcriptome-wide methods for RNA editing detection. Furthermore, deep sequencing based approaches allowed to identify a large number of editing sites, up to two orders of magnitude higher than before. Two years later, a new release of DARNED recorded more than 330,000 editing sites in human (Kiran et al., 2013). This led to the design of tools to both visualize and annotate RNA-Seq data with known editing sites (Picardi et al., 2011; Distefano et al., 2013).

Although DARNED contains precious information regarding known editing sites, only a small portion of this have been later manually annotated, not providing any information about the spatiotemporal regulation of editing events through their editing level (Wahlstedt et al., 2009; Solomon et al., 2014). To improve this aspect, Ramaswami and Li built RADAR^4^, a rigorously annotated database of A-to-I editing sites. Particularly, they have enriched RNA editing knowledge by including detailed manually curated information for each editing site, such as genomic coordinates, type of genomic region (intergenic region, 3′-or-5′ UTR, intron, or coding sequence if the editing site occurs in genic region), type of repetitive element (when the editing event occurs in Alu or not-Alu element), the conservation in other species (chimpanzee, rhesus, mouse), and the tissue-specific editing level when known. Currently, RADAR contains about 1.4 million editing sites as detected in Homo Sapiens (Ramaswami and Li, 2014). Among them, the editing sites that occur in human ncRNAs are only a small fraction, consisting of about 21,000 events, with only 1,219 editing sites in microRNAs. Despite being a relatively small percentage, amounting to about 1.6% of the total number of human editing sites, these miRNA editing events may very well posses significant importance as far as the editing phenomenon is concerned.

Without a doubt, continuous updating of the RADAR database gradually will become a precious resource for researchers in this field, leading to a better understanding of the editing phenomenon in coming years.

### Effect of RNA editing in non-coding RNA molecules

**Figure 1:**
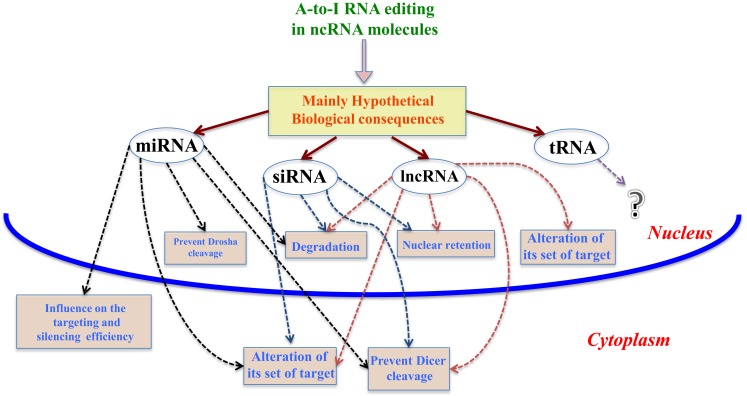
**Mainly hypothetical biological consequences**. In this figure, we show some of the main biological consequences of A-to-I RNA editing in ncRNA molecules, both in nucleus and cytoplasm.

In the last decade, editing events have been discovered in ncRNA molecules, such as miRNAs, siRNAs, tRNAs, and lncRNAs. Although not fully demonstrated yet, these editing sites could alter the stability, the biogenesis, and target recognition of ncRNAs, as shown in Figure 1.

#### RNA editing in miRNAs and siRNAs

As seen above, many A-to-I editing sites in miRNAs have been discovered (Luciano et al., 2004; Kawahara et al., 2007; Alon et al., 2012), and these could influence miRNA-mediated gene regulation in several ways (Nishikura, 2010), although in some cases low percentage editing of mature miRNAs could be a low level of genomewide editing noise rather than possessing biological relevance (de Hoon et al., 2010). First, editing sites occurring in pri-miRNAs can suppress cleavage processing by Drosha and/or Dicer due to the presence of inosines, while in addition, highly edited dsRNAs could be rapidly degraded by Tudor-SN (TSN) (Yang et al., 2006). Second, some editing events in pri-miRNAs can produce edited pre-miRNAs, for which different scenarios can occur based on the location of the editing site. In particular, studies have demonstrated that A-to-I editing sites in miRNA seed regions can drastically change their target set (Kawahara et al., 2008; Alon et al., 2012), causing a functional transformation, but also affect the mRNA target selection and silencing processes (Kume et al., 2014).

Small interfereing RNAs, differently from miRNAs, originate from long double-strand RNAs exported to the cytoplasm, where they are cleaved by the Dicer-TRBP complex and successively loaded inside the RISC complex. It has been observed that ADAR1-p150, which acts in the cytoplasm, can bind to siRNAs preventing and thus overall reducing the cleavage process of the Dicer-TRBP complex (Yang et al., 2005; Kawahara et al., 2007).

Lately, a new role for ADAR1-p150 not associated to RNA editing was discovered, in which the enzyme forms an heterodimer complex with Dicer by protein–protein interaction (PPI), increasing the rate of siRNA and miRNA processing and facilitating RISC loading and RNA silencing, instead of an antagonistic role in RNAi by an ADAR1–ADAR1 homodimer complex (Nishikura et al., 2013; Ota et al., 2013).

#### RNA editing in lncRNAs

Another category of ncRNAs is represented by long non-coding RNAs (lncRNAs). In recent years, HTS analyses have led to the identification of thousands of lncRNAs, many of which have revealed to be transcripts deriving from the antisense strand of protein coding genes. lncRNAs, due to their stable long double-strand regions, often originating from the presence of repetitive elements, such as Alus, can be affected by A-to-I RNA editing (Peng et al., 2012). The biological functions of A-to-I editing occurring in lncRNAs can be several.

Long non-coding RNAs can be retained in the nucleus as a consequence of the editing phenomenon until cleavage of the hyper-edited region takes place and the remaining lncRNA portion is exported to the cytoplasm (Prasanth et al., 2005). Nevertheless, as for miRNAs (Yang et al., 2006), edited lncRNAs could though be degraded through Tudor-SN. Considering the property lncRNAs possess to bind with RNA and DNA (Rinn and Chang, 2012; Mercer and Mattick, 2013), as well as RNA binding proteins (Hellwig and Bass, 2008), cases of editing sites in lncRNAs could clearly change their target set and RNP structures respectively, thus altering their intrinsic biological function (Geisler and Coller, 2013). Finally, a far more rare RNA editing phenomenon compared to the one caused by inverted repeat structures in mRNAs could occur for those transcripts which associate to antisense lncRNAs, providing a double strand RNA structure suitable for ADAR as suggested in (Geisler and Coller, 2013).

#### RNA editing in tRNAs

Differently from mRNAs and several categories of ncRNA molecules which undergo A-to-I editing primarily by ADARs, A-to-I editing events in mature transfer RNAs (tRNAs) in eukaryotes, can possibly be a result of adenosine deaminases acting on tRNA enzyme family (ADATs) (Su and Randau, 2011). A-to-I editing in these small ncRNAs is conserved in various species and occurs principally at positions 34, 37, and 57 of certain tRNAs (Torres et al., 2014). Despite this phenomenon being ubiquitously present in human tissues, the role of A-to-I tRNA editing remains still unknown.

## Conclusion

As seen above, currently Alon’s pipeline is the only HTS-based method to systematically identify A-to-I editing sites in pre- and mature microRNAs. There is a current and urgent necessity for new HTS-based methodologies to emerge in order to not only accurately identify and analyze editing events in other categories of ncRNA molecules, such as tRNAs, lncRNAs, and so on, but also to investigate through functional enrichment analysis, the biological outcomes that a single editing event can generate. Concurrently, it could be interesting to analyze how the editing phenomenon can influence a biological pathway within a temporally changing cellular condition, such as starvation or hypoxia, considering that a single editing site in a ncRNA molecule could drastically modify its function.

## Conflict of Interest Statement

The authors declare that the research was conducted in the absence of any commercial or financial relationships that could be construed as a potential conflict of interest.

## Footnotes

^1^http://www.biochem.utah.edu/bass/index.html

^2^http://darned.ucc.ie

^3^http://microrna.osumc.edu/mireditar

^4^http://RNAedit.com
